# Full Counting Statistics of Electrons through Interaction of the Single Quantum Dot System with the Optical Field

**DOI:** 10.3390/nano9030394

**Published:** 2019-03-08

**Authors:** Weici Liu, Faqiang Wang, Zhilie Tang, Ruisheng Liang

**Affiliations:** 1 Guangdong Research Center of Photoelectric Detection Instrument Engineering Technology, and Guangdong Laboratory of Quantum Engineering and Quantum Materials, School of physics and Telecommunication Engineering, South China Normal University, Guangzhou 510006, China; liuweici-2002@126.com; 2 Department of Electronic Information Engineering, Guangzhou College of Technology and Business, Foshan 528138, China; 3 Guangzhou Key Laboratory for Special Fiber Photonic Devices, Laboratory of Nanophotonic Functional Materials and Devices, School of Information and Optoelectronic Science and Engineering, South China Normal University, Guangzhou 510006, China; fqwang@scnu.edu.cn (F.W.); gdz01@scnu.edu.cn (R.L.)

**Keywords:** quantum dot, full counting statistics, particle-number-resolved master equation, optical fields

## Abstract

In this paper, using the particle-number-resolved master equation, the properties of full counting statistics (FCS) are investigated for a single quantum dot (QD) system interacting with optical fields in the thermal state, Fock state, coherent state, and coherent state with random phase. In these diverse quantum states of optical fields, average tunneling currents have different step shoulder heights at a lower bias voltage with the same light intensity, and a staircase-shaped current can be induced unexpectedly in vacuum state optical field. The characteristics of the Fano factor and skewness in the coherent state differ from those in all of the other cases. For avalanche-like transport at a lower bias voltage, the mechanism is a dynamical channel blockade in a moderate electron–photon interaction regime. There is a pronounced negative differential conductance that results from tuning the phase of the coherent state optical field in a symmetric QD system.

## 1. Introduction

The full counting statistics (FCS) of charge transfer in quantum dots (QDs), molecules, and nanostructures are an important component in the characterization of microscopic transport processes because the FCS contains complete information about low-frequency current fluctuations [1]. Theoretically, FCS can provide all higher-order moments of current fluctuations and offer deep insight into the nature of transport mechanisms not encoded in the first two moments [2]. In particular, the system parameters dominating the transport can be extracted from current-noise measurements. Experimentally, real-time counting statistics have been calculated for single-electron tunneling transport through QDs, and this is a crucial achievement that enables counting individual electron tunnel events [3]. Until now, FCS have been used to study different systems, such as a mesoscopic superconductor [4], a voltage-biased superconducting nano-junction [5], multichannel chaotic cavities [6], a ballistic chaotic cavity [7], a coupled QD system [8], single-molecule junctions [9], and atomic spin devices [10].

On the other hand, owing to the versatility of nanofabricated circuits, hybrid circuit quantum electrodynamics (QED) can provide numerous diverse situations that are not accessible with the standard cavity QED [11]. The advantage of hybrid circuit QED systems is that the properties of a nanostructure device can be arbitrarily controlled by an externally applied field. QD systems can be used in circuit QED to probe light–matter interactions [12,13,14,15], implement a QD laser [16,17], and engineer new states of matter relevant to solid-state physics [18]. In addition to that, the QD is one of the candidates for use as a quantum information device [19]. 

In an effort to construct quantum optical and quantum electronic QD devices, photon-assisted electronic transport has been investigated by many scientists [20]. However, to study the current and shot noise, most of the studies have employed the classical treatment of the external field, which introduces a time-dependent oscillating energy level in the QD [20,21]. Furthermore, the results are the same if the intensities of the different external optical fields are the same. The classical treatment for photons is valid at high field intensity and for the case of weak coupling in an equilibrium system, but it does not work in the vacuum state or other quantum states of light [22]. Moreover, there are many papers that discuss the FCS properties of a single-molecule junction, which has a similar Hamiltonian (QD interacting with a phonon) to that presented in this paper [23,24]. In previously published papers, the phonon distribution is either in equilibrium or nonequilibrium. The phonon distribution is a dominant factor in determining the transport properties. Usually, a molecule attached to its substrate exhibits an equilibrium phonon distribution while the phonons of a molecule air-bridged to electrodes have a nonequilibrium distribution [25]. The equilibrium state of the aforementioned phonon is a thermal state. In this paper, we apply a Hamiltonian with the quantum treatment of an optical field to study the influences of different quantum states of optical fields (thermal state, Fock state, coherent state, and coherent state with random phase) on the FCS properties of a QD in which the photon distribution is in equilibrium.

The method used in this paper is the particle-number-resolved master equation [26,27]. An alternative method is to use rate equations to derive the tunneling rate by Fermi’s golden rule, in which the term that describes the relaxation of vibrations toward the equilibrium distribution is added by hand [23,24,28]. In rate equations, all off-diagonal components are ignored because they decay rapidly in certain parameter regimes [29].

This paper is organized as follows. In Section 2, we present the physical model and theoretical research by the particle-number-resolved master equation approach. In Section 3, we study the influences of optical fields with different statistical properties on the average current, Fano factor, and skewness of single-electron tunneling for different parameters. The conclusion is given in Section 4. 

## 2. Physical Model and Formalism

**Figure 1:**
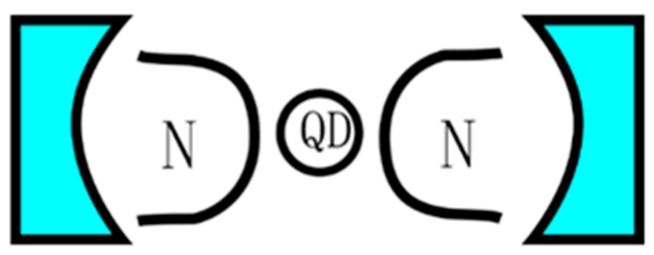
The system structure sketch.

Figure 1 illustrates the system schematic of the single QD with normal electrode leads coupled to a one-mode optical cavity. The QD is modeled as a one-level system. The loss of the optical field is not considered here because our focus is on the influence of different optical fields on the transport through a single QD.

The total Hamiltonian of the system can be written as *H* = *H_L_* + *H_R_* + *H_ph_* + *H_D_* + *H_T_*. The Hamiltonian for electrons in the left (*L*) and right (*R*) electrode leads are (e=ℏ=1) [22,23,24]

(1) $$H_{L}+H_{R}=\sum_{k,\alpha \in L,R}\varepsilon_{k,\alpha }c_{k,\alpha }^{†}c_{k,\alpha }$$

where $c_{k,\alpha }^{†}(c_{k,\alpha })$ is the conduction electron creation (annihilation) operator with wave vector *k* in electrode lead α, and $\varepsilon_{k,\alpha }$ is the single-electron energy. The third term *H_ph_* stands for the single-mode optical field, and $H_{ph}=\omega_{0}a^{†}a$, where $a^{†}\left(a\right)$ is the photon creation (annihilation) operator with frequency $\omega_{0}$. The electron Hamiltonian on the QD is

(2) $$H_{D}=\left[\varepsilon +eV_{g}+\lambda (a^{†}+a)\right]d^{†}d$$

where ε denotes the energy level of the dot and can be controlled by modulating the gate voltage $V_{g}$, and λ is the coupling constant between the dot electron and photon mode. $d^{†}\left(d\right)$ is the corresponding creation (annihilation) of an electron on the QD, and we assume the on-site Coulomb repulsion on the QD is infinite. The last term *H_T_* describes the conduction electron hopping between the QD and electrode leads,

(3) $$H_{T}=\sum_{k,\alpha \in L,R}T_{k,\alpha }c_{k,\alpha }^{†}d+H.c.$$

where $T_{k,\alpha }$ is a tunneling matrix element.

It is convenient to eliminate the electron–photon coupling terms in the Hamiltonian by a canonical transformation, i.e., $\tilde{H}=e^{s}He^{-s}$ with $s=\left(a^{†}-a\right)d^{†}d$ and $=\lambda /\omega_{0}$, so the transformed Hamiltonian becomes $\tilde{H}={\tilde{H}}_{el}+{\tilde{H}}_{sys}$, where ${\tilde{H}}_{sys}=\omega_{0}a^{†}a+\tilde{\varepsilon }d^{†}d$, and

(4) $${\tilde{H}}_{el}=\sum_{k,\alpha \in L,R}\varepsilon_{k,\alpha }c_{k,\alpha }^{†}c_{k,\alpha }+\sum_{k,\alpha \in L,R}\left(XT_{k,\alpha }c_{k,\alpha }^{†}d+H.c.\right)$$

where $\tilde{\varepsilon }=\varepsilon -\delta +V_{g}$, $\delta =g\omega_{0}$, $g{=}^{2}$, $X=\exp\left[-\left(a^{†}-a\right)\right]$.

To investigate an open quantum system, such as a single QD interacting with an optical field and coupled to a reservoir of electrons in the left and right electrode leads, it is appropriate to employ the reduced density matrix of this system ρ, which is obtained by taking the trace over the degrees of freedom of the reservoirs and optical field [27,30],

(5) $$\frac{d}{dt}\rho =-i[{\tilde{H}}_{sys},\rho ]-\int_{0}^{\infty }dt^{’}Tr_{leads+opt}\left\{\left[{\tilde{H}}_{T},\left[{\tilde{H}}_{T}(-t^{’}),\rho \right]\right]\right\}⊗\rho_{opt}⊗\rho_{leads}$$

Then, we can get the Born–Markov master equation as

(6) $$\begin{array}{cc} \frac{d}{dt}\rho = & -i[{\tilde{H}}_{sys},\rho ]+(W_{1}^{R}+W_{3}^{R})D\left[d^{†}\right]\rho +(W_{2}^{R}+W_{4}^{R})D\left[d\right]\rho -\\ & i(W_{2}^{I}+W_{4}^{I}+W_{1}^{I}+W_{3}^{I})\left[\rho ,d^{†}d\right]/2 \end{array}$$

where the notation D is defined as $D\left[A\right]\rho =A\rho A^{†}-\frac{1}{2}\left[A^{†}A\rho +\rho A^{†}A\right]$, and the superscript R and I represent the real part and imaginary part, respectively. $W_{i}$ (i = 1, 2, 3, 4) represents the tunneling rate between the right (left) leads and the QD [27,30].

(7a) $$W_{1}=\int \frac{d\omega }{2\pi }f_{L}(\tilde{\varepsilon }+\omega )F^{<}(\omega )\Gamma_{L}$$

(7b) $$W_{2}=\int \frac{d\omega }{2\pi }[1-f_{L}(\tilde{\varepsilon }+\omega )]F^{>}(\omega )\Gamma_{L}$$

(7c) $$W_{3}=\int \frac{d\omega }{2\pi }f_{R}(\tilde{\varepsilon }+\omega )F^{<}(\omega )\Gamma_{R}$$

(7d) $$W_{4}=\int \frac{d\omega }{2\pi }[1-f_{R}(\tilde{\varepsilon }+\omega )]F^{>}(\omega )\Gamma_{R}$$

where $\Gamma_{\alpha }=2\pi \rho_{\alpha }\sum_{k}{\left|T_{k,\alpha }\right|}^{2}$, $f_{\alpha }$ is the Fermi distribution function for lead α, and $F^{>\left(<\right)}\left(\omega \right)$ is the Fourier transform of the bosonic correlation function $\langle X^{†}\left(t\right)X\rangle$ ($\langle X\left(t\right)X^{†}\rangle$). We employ the standard decoupling approximation, which assumes that the averages of the products of two operators ($\langle X^{†}\left(t\right)X\rangle$ and $\langle X\left(t\right)X^{†}\rangle$) can be evaluated in the equilibrium state. This approximation, called the single-particle approximation [31], is valid if $\omega_{0}$ or *λ* is large compared with the QD-lead coupling [32]. After expanding the exponents in a power series and applying the Cauchy product to the power series, we can obtain [33,34]

(8a) $$F_{thermal}^{<}(\omega )=\sum_{n=-\infty }^{\infty }\exp\left[-g\left(1+2N_{th}\right)\right]{\left(\frac{1+N_{th}}{N_{th}}\right)}^{\frac{n}{2}}\times I_{n}\left(2g\sqrt{N_{th}\left(1+N_{th}\right)}\right)\delta (\omega -n\omega_{0})$$

(8b) $$F_{thermal}^{>}(\omega )=\sum_{n=-\infty }^{\infty }\exp\left[-g\left(1+2N_{th}\right)\right]{\left(\frac{1+N_{th}}{N_{th}}\right)}^{\frac{n}{2}}\times I_{n}\left(2g\sqrt{N_{th}\left(1+N_{th}\right)}\right)\delta (\omega +n\omega_{0})$$

for an optical field in a thermal state, $\rho_{opt}=\sum_{N=0}^{\infty }\frac{N_{th}^{N}}{{\left(1+N_{th}\right)}^{N+1}}|N\rangle \langle N|$, which is a field emitted by a classical light source in thermal equilibrium at temperature *T* [35]; |N〉 is the eigenstate of the photon particle number operator $a^{†}a$.

(9a) $$F_{Fock}^{<}(\omega )=e^{-\zeta^{2}}\sum_{l=0}^{N_{Fock}}\sum_{k=0}^{\infty }\frac{N_{Fock}!\zeta^{2l}{\left(-1\right)}^{k}L_{k}^{2l-k}(\zeta^{2})}{l!l!\left(N_{Fock}-l\right)!}\delta (\omega -(k-l)\omega_{0})$$

(9b) $$F_{Fock}^{>}(\omega )=e^{-\zeta^{2}}\sum_{l=0}^{N_{Fock}}\sum_{k=0}^{\infty }\frac{N_{Fock}!\zeta^{2l}{\left(-1\right)}^{k}L_{k}^{2l-k}(\zeta^{2})}{l!l!\left(N_{Fock}-l\right)!}\delta (\omega +(k-l)\omega_{0})$$

for an optical field in a Fock state, $|N_{Fock}\rangle$, which can be generated by a cavity quantum electrodynamics scheme using the strong coupling between excited atoms and a single-mode cavity field [36]. The number of photons is exactly defined for a Fock state.

(10a) $$F_{coherent}^{<}(\omega )=e^{-\zeta^{2}+\zeta \alpha^{*}-\zeta \alpha }\sum_{n=-\infty }^{\infty }{\left(\sqrt{\frac{\zeta +\alpha }{\alpha^{*}}}\right)}^{n}J_{n}\left(2\zeta \sqrt{\zeta \alpha^{*}+{\left|\alpha \right|}^{2}}\right)\delta (\omega -n\omega_{0})$$

(10b) $$F_{coherent}^{>}(\omega )=e^{-\zeta^{2}+\zeta \alpha -\zeta \alpha *}\sum_{n=-\infty }^{\infty }{\left(\sqrt{\frac{\zeta -\alpha^{*}}{\alpha }}\right)}^{n}I_{n}\left(2\zeta \sqrt{\zeta \alpha -{\left|\alpha \right|}^{2}}\right)\delta (\omega +n\omega_{0})$$

for an optical field in a coherent state, |α〉, which is the eigenstate of the photon annihilation operator *a* and has a more precisely defined phase. A coherent state is the closest quantum approximation to the field generated by a laser. A coherent state does not contain a definite number of photons, and this follows from the fact that a coherent state is made up of a coherent superposition of Fock states, $|\alpha \rangle =\sum_{n=0}^{\infty }|n\rangle \langle n|\alpha \rangle$. Here, $I_{n}$ is the nth modified Bessel function, and $J_{n}$ is the nth first kind of Bessel function.

(11a) $$F_{Random}^{<}(\omega )=e^{-{\left|\alpha \right|}^{2}}\sum_{n=-\infty }^{\infty }\frac{{\left|\alpha \right|}^{2n}}{n!}F_{Fock}^{<}(\omega )$$

(11b) $$F_{Random}^{>}(\omega )=e^{-{\left|\alpha \right|}^{2}}\sum_{n=-\infty }^{\infty }\frac{{\left|\alpha \right|}^{2n}}{n!}F_{Fock}^{>}(\omega )$$

if the optical field is in a coherent state with random phase, $\rho_{opt}=e^{-{\left|\alpha \right|}^{2}}\sum_{n=0}^{\infty }\frac{{\left|\alpha \right|}^{2n}}{n!}|n\rangle \langle n|$ [37]. Here, $L_{k}^{2l-k}$ represents Laguerre polynomials. If we stay within the coherent length of the laser and work at frequencies for which the laser’s intensity noise is at a quantum noise limit, then a laser beam can represent an excellent realization of an ideal coherent state [38]. Otherwise, it represents a random phase coherent state.

Because we assume that the Coulomb repulsion on a QD is infinite, there are only two states for a QD: an unoccupied state and an occupied state. Denoting 〈0|ρ|0〉 and 〈1|ρ|1〉 as $\rho_{0}$ and $\rho_{1}$, respectively, then, using Equation (6), one can obtain the particle-number-resolved master equation,

(12a) $$\frac{d}{dt}\rho_{0}^{n}=-(W_{1}^{R}+W_{3}^{R})\rho_{0}^{n}+W_{2}^{R}\rho_{1}^{n}+W_{4}^{R}\rho_{1}^{n-1}$$

(12b) $$\frac{d}{dt}\rho_{1}^{n}=-(W_{2}^{R}+W_{4}^{R})\rho_{1}^{n}+W_{1}^{R}\rho_{0}^{n}+W_{3}^{R}\rho_{0}^{n+1}$$

Then, the particle-number-resolved master equation can be rewritten as

(13) $$\frac{d}{dt}\rho^{n}=A\rho^{\left(n\right)}+B\rho^{\left(n+1\right)}+C\rho^{\left(n-1\right)}$$

where $A=\left[\begin{array}{l} -(W_{1}^{R}+W_{3}^{R}),W_{2}^{R}\\ W_{1}^{R}\;,-(W_{2}^{R}+W_{4}^{R}) \end{array}\right]$ $B=\left[\begin{array}{l} \;0\;,0\\ W_{3}^{R},0 \end{array}\right]$ $C=\left[\begin{array}{l} 0,W_{4}^{R}\\ 0,0 \end{array}\right]$.

In order to facilitate the calculation of FCS, we introduce the following matrix S(χ,t), which is defined by $S\left(\chi ,t\right)=\sum_{n}\rho^{n}\left(t\right)e^{in\chi }$, where χ is the counting field [21]. Since the particle-number-resolved master equation has the form given in Equation (13), S(χ,t) satisfies the equation

(14) $$\frac{d}{dt}S=L_{\chi }S,\;L_{\chi }=A+Be^{-i\chi }+Ce^{i\chi }$$

where

(15) $$L_{\chi }=\left[\begin{array}{l} -(W_{1}^{R}+W_{3}^{R}),\;W_{2}^{R}+W_{4}^{R}e^{i\chi }\\ W_{1}^{R}+W_{3}^{R}e^{-i\chi },-(W_{2}^{R}+W_{4}^{R}) \end{array}\right]$$

The cumulant generating function (CGF) is defined as $e^{-F\left(\chi \right)}=Tr\left[S\left(\chi ,t\right)\right]$. Thus, under the low-frequency limit, F(χ) can be written as F(χ)=−λ(χ)t, where λ(χ) is the eigenvalue of $L_{\chi }$ and tends to zero (λ(χ)→0) as χ→0 [26]. Using Equation (15), one can get

(16) λ(χ)=1t∑k=1∞Ck(iχ)kk!=1t{C1χ−C2χ22−iC3χ36+…}

which can be substituted into

(17) $$\left|L_{\chi }-\lambda I\right|=0$$

After some algebra, one can obtain the first three cumulants, *C*_1_, *C*_2_, and *C*_3_, as

(18a) $$\frac{C_{1}}{t}=\frac{W_{1}^{R}W_{4}^{R}-W_{2}^{R}W_{3}^{R}}{W_{1}^{R}+W_{2}^{R}+W_{3}^{R}+W_{4}^{R}}$$

(18b) $$\frac{C_{2}}{t}=\frac{1}{W_{1}^{R}+W_{2}^{R}+W_{3}^{R}+W_{4}^{R}}\left\{W_{1}^{R}W_{4}^{R}+W_{2}^{R}W_{3}^{R}-2{\left(\frac{C_{1}}{t}\right)}^{2}\right\}$$

(18c) $$\frac{C_{3}}{t}=\frac{1}{W_{1}^{R}+W_{2}^{R}+W_{3}^{R}+W_{4}^{R}}\left\{W_{1}^{R}W_{4}^{R}-W_{2}^{R}W_{3}^{R}-6\frac{C_{1}}{t}\frac{C_{2}}{t}\right\}$$

The first-order cumulant, defined as the average of the electron number distribution C_1_ = *n*(*t*), is clearly related to the average current, *I* = *eC*_1_/*t*. The second-order cumulant $C_{2}=\bar{n^{2}}-{\bar{n}}^{2}$ is the mean-square deviation and is related to the zero-frequency shot noise, defined as *S* = 2*e*^2^*C*_2_/*t*. The third cumulant $C_{3}=\bar{{\left(n-\bar{n}\right)}^{3}}$ characterizes the skewness of the distribution. The overbar denotes the statistical average, that is $\left(\cdots \right)=\sum_{n}\left(\cdots \right)P\;\left(n,t\right)$. In general, the normalized second-order cumulant *F_a_* = *C*_2_/*C*_1_ is called the Fano factor, which is the shot noise, and the third cumulant *S_k_* = *C*_3_/*C*_1_ is used to describe the skewness. The electron-number-resolved statistic is called the super-Poissonian for *F_a_* > 1 and sub-Poissonian for *F_a_* < 1.

## 3. Discussion

In the following discussion, we set the effective energy level to $\tilde{\varepsilon }=E_{0}=E_{F}+V_{g}-\delta$, with $E_{F}=0$, which is the equilibrium chemical potential of the left (right) electrode lead in the absence of gate voltage $V_{g}$; $\mu_{L}$($\mu_{R}$) is the chemical potential of the left (right) electrode lead, which is taken as $\mu_{L\left(R\right)}=E_{F}\pm V_{bias}/2$; and $V_{bias}$ is the bias voltage between two electrode leads. We also assume the tunneling rate $\Gamma_{\alpha }$ as $\Gamma_{l}=\Gamma_{r}=\Gamma_{0}=0.1$, with the photon frequency $\omega_{0}=1$ $\left(\Gamma_{0}\ll \omega_{0}\right)$. The energy unit is taken as the photon frequency $\omega_{0}$ throughout this paper.

Now, we begin to discuss the influences of different types of optical fields on the FCS properties of charge transport on QDs. 

**Figure 2:**
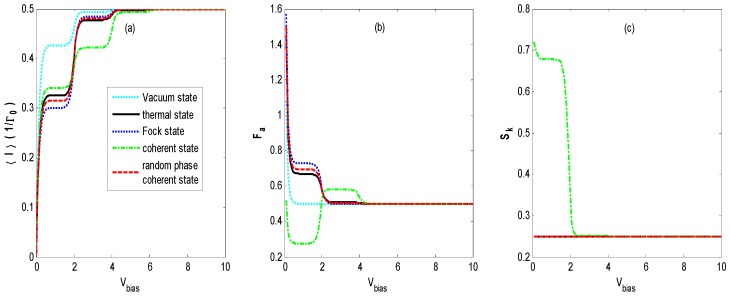
〈I〉 (**a**), *F_a_* (**b**) and *S_k_* (**c**) versus the bias voltage *V_bias_* for different states of optical field with photon intensity *N_th_* = *N_Fock_* = $\alpha^{2}$ = 1, electron–photon coupling constant *λ* = 0.4, effective QD energy level $\tilde{\varepsilon }=0$, and the lead’s temperature $\beta =1/K_{B}T=20$. The skewness curves coincide for the vacuum state, thermal state, Fock state, and coherent state with random phase.

**Figure 3:**
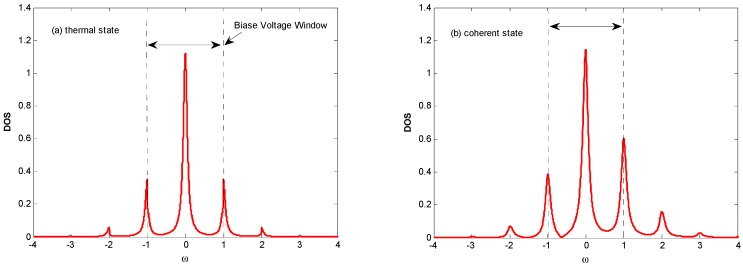
Density of state for quantum dot electron coupling with the thermal state (**a**) and coherent state (**b**) optical fields, respectively. It is a schematic diagram for the principle of the dynamical channel blockade.

Figure 2a shows the average tunneling current as a function of *V_bias_* in the regime of moderate electron-photon coupling (ζ<1). In Figure 2a, the well-known staircase-shaped current appears for different states of optical fields. As *V_bias_* increases to cover the photon sidebands, the photon-assisted resonant tunneling channels open one by one, leading to an increasing number of current steps with decreasing shoulder heights, as presented in Figure 3. This is similar to the results of the electron–phonon coupling case [9,23,24,25] or the AC-driven nanostructure system [20,21]. The average tunneling current has turning points at bias voltages of *V_bias_* = 2*n*$\omega_{0}$ because the photon sidebands locate at $n\omega_{0}\left(n=\pm 1,\pm 2,\ldots \ldots \right)$, which is understandable from Equations (7)–(11). When the light intensity is the same for all of the quantum states of optical fields, the average tunneling currents have the same saturation current at a higher bias voltage *V_bias_*, but they have different shoulder heights at a lower bias voltage *V_bias_* in Figure 2. Our result is different from that obtained by the classical treatment of the photon field in which currents are the same if the intensities of different external optical fields are identical [20,21].

Furthermore, we find that the vacuum state optical field also induces a staircase-shaped average current (cyan dotted line). That means that the photon sidebands still work in the quantum case because of the interaction of the QD’s electron with the vacuum fluctuations, which is undiscovered in the case of an AC-driven nanostructure system [20,21]. Comparing the curve of the vacuum state with those of the other states, we can observe that the average current is markedly suppressed as the photon intensity increases from zero to one.

Figure 2b demonstrates that the Fano factor-voltage characteristics exhibit a singularity at zero bias voltage, which corresponds to the finite thermal Nyquist-Johnson noise due to the thermal fluctuations in the occupation number in the left and the right leads [2]. It can be seen that the Fano factor in the vacuum state (cyan dotted line) decreases quickly to 0.5 with an increasing bias voltage *V_bias_*. This limit corresponds to the regime of the Pauli blockade and Coulomb blockade, in which electron transport is anticorrelated due to the Coulomb repulsion and the Pauli exclusion principle [39,40]. The Fano factor-voltage characteristics for the thermal state, Fock state, and coherent state with random phase are almost the same and decrease to 0.5 with two steps with increasing bias voltage *V_bias_*. However, for the coherent state, as the bias voltage *V_bias_* increases, *F_a_* decreases to about 0.3, then rises abruptly to about 0.6, and finally decreases to 0.5. This pattern is different from those in other optical fields.

The staircase-shaped Fano factor in Figure 2b can be interpreted by the mechanism termed the *dynamical channel blockade* [41]. In order to explain the principle, we provide a schematic diagram in Figure 3, which gives the density of the state for a QD’s electron coupling with the thermal state and coherent state optical fields, respectively. From Figure 3, one can find that there are photon sidebands located at $n\omega_{0}\left(n=\pm 1,\pm 2,\ldots \ldots \right)$ resulting from the interaction of an electron on the QD with the optical field, which is similar to that of the QD system interacting with a phonon [42]. Once an electron jumps to a QD from an electrode lead, it can jump on the photon sideband outside the bias voltage window by absorbing a photon. Because of the Coulomb blockade and Pauli blockade, no other electrons can jump to the QD, and this leads to the dynamical channel blockade due to the small tunneling rate of the occupied channel. As the bias voltage increases, the dynamical channel blockade can be gradually removed if all the photon sidebands enter the bias voltage window. Thus, the Fano factor will walk into the regime dominated by the Pauli blockade and Coulomb blockade from the regime dominated by the dynamical channel blockade with an increasing bias voltage. 

From Figure 2b, it can also be found that the Fano factor for the coherent state is less than that of the vacuum state for a small bias voltage. The reason is that $F_{coherent}^{<}$ and $F_{coherent}^{>}$ (Equation (10)) contain contributions from the off-diagonal terms of $\langle n\left|X\left(t\right)X^{†}\right|m\rangle$ and $\langle n|X^{†}X\left(t\right)|m\rangle$ (n≠m), respectively, in the photon number representation. This will make the amplitude of the photon sideband of the coherent state fall below that of the zero-field vacuum contribution for certain ranges of parameters. Thus, the coherent state could suppress the tunneling contribution of the vacuum state for certain ranges of parameters [22].

**Figure 4:**
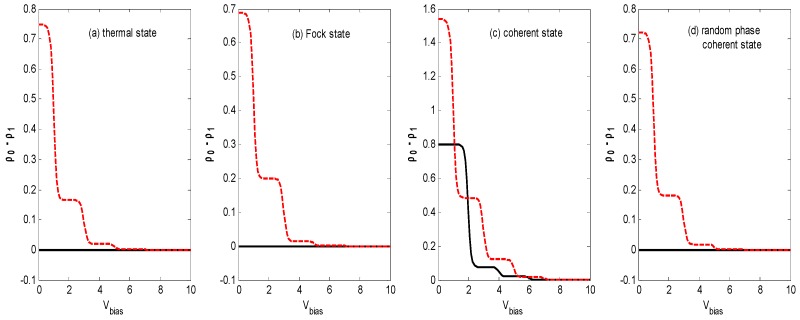
$\rho_{0}-\rho_{1}$ versus the bias voltage *V_bias_* for different states of optical field with the same parameters as Figure 2. Solid dark curves for $\tilde{\varepsilon }=0$; dashed red curves for $\tilde{\varepsilon }=0.5$. (**a**) thermal state, (**b**) Fock state, (**c**) coherent state and (**d**) random phase coherent state.

Figure 2c exhibits the skewness-voltage characteristics for the vacuum state, thermal state, Fock state, coherent state, and coherent state with random phase. The *S_k_* for the coherent state decreases to about 0.7, then steps down to a constant value that is the same as that for the other quantum states with an increasing bias voltage V_bias_, but it remains a constant value for the other three quantum states as the bias voltage *V_bias_* increases. In order to explain the skewness-voltage characteristics, the curve of $\rho_{0}-\rho_{1}$ versus the bias voltage of a steady solution is plotted for all four quantum states. The solid dark curves correspond to the same parameters as in Figure 2. Comparing the corresponding curves in Figure 2c and Figure 4, there seems to be a correlation between skewness and $\rho_{0}-\rho_{1}$.

**Figure 5:**
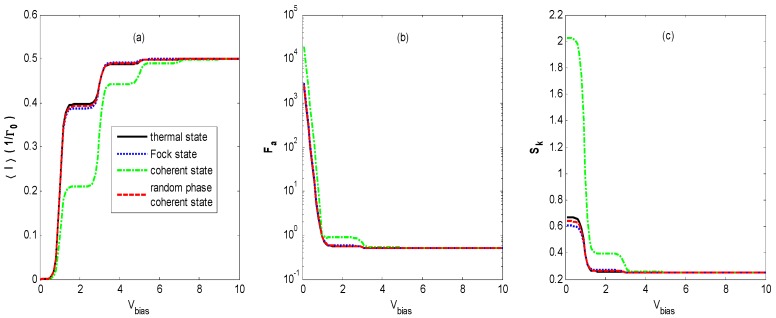
〈I〉 (**a**), *F_a_* **(b**), and *S_k_* (**c**) versus the bias voltage *V_bias_* for different states of optical field with the same parameters as Figure 2 except for the effective energy level of the QD, $\tilde{\varepsilon }=0.5$. The Fano factor curves coincide for the thermal state, Fock state, and coherent state with random phase.

Figure 5 shows the FCS properties of QD charge transport versus the bias voltage *V_bias_* for different states of optical field for the same parameters as shown in Figure 2, except the effective energy level of the QD is shifted to 0.5. From Figure 5a, it can be observed that the first step of the current is shifted due to the shift of the effective energy level of the QD. Figure 5a also exhibits that the current–voltage characteristics for the thermal state, Fock state, coherent state, and coherent state with random phase are almost the same as those in Figure 2a. Because the effective energy level of the QD is shifted away from the main channel and the photon sideband channel, there are no average currents when the bias voltage starts to increase. The shoulder height of the first and third steps of the current becomes smaller, and it becomes bigger for the second step for the coherent state. 

Figure 5b demonstrates that the Fano factor–voltage characteristics are different from that in Figure 2b. The Fano factors are at least several orders of magnitude higher than those in Figure 2b at lower bias voltage, and the Fano factor in the coherent state decreases to 0.5 with one step as bias voltage *V_bias_* increases, and the step is not obvious for the thermal state, Fock state, and coherent state with random phase compared with that in Figure 2b. This super-Poissonian value of the Fano factor (*F_a_* > 1) is due to avalanche-like transport [24]. Because the effective energy level of the QD is shifted away from the main channel and the photon sideband channel, the dynamical channel blockade arises at the beginning of the increase in the bias voltage, which results in avalanche-like transport for the four quantum states of optical field. The mechanism for avalanche-like transport in this paper is the dynamical channel blockade for a moderate electron–photon interaction regime (λ<1), while the mechanism for avalanche-like transport in Reference [24] is one in which the transitions between low-lying phonon states are exponentially suppressed for strong electron-phonon coupling (λ≫1). Moreover, the photon distribution described in this paper is in equilibrium, while the phonon distribution is in a nonequilibrium distribution in Reference [24].

Figure 5c demonstrates that the skewness-voltage characteristic for the coherent state is almost the same as that in Figure 2c, except that it has one more step before it decreases to a constant value. Compared with that in Figure 2c, at a lower bias voltage, the curve in Figure 5c has two steps before it decreases to a constant value for the thermal state, Fock state, and coherent state with random phase. Comparing the curves in Figure 5c with the dashed red curves in Figure 4, the correlation between skewness and $\rho_{0}-\rho_{1}$ is apparent.

**Figure 6:**
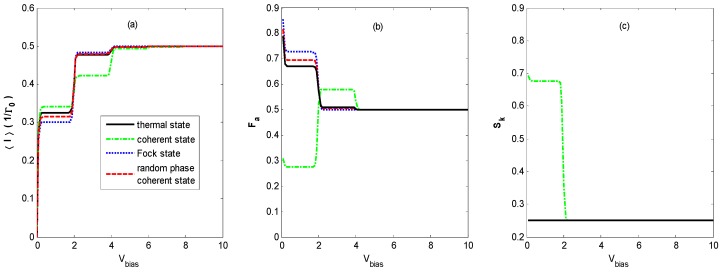
〈I〉 (**a**), *F_a_* (**b**), and *S_k_* (**c**) versus the bias voltage *V_bias_* for different states of optical field with the same parameters as Figure 2 except for the lower temperature, β=50. The skewness curves coincide for the thermal state, Fock state, and coherent state with random phase.

Figure 6 shows the FCS properties of QD charge transport versus the bias voltage *V_bias_* for different states of optical field for the same parameters as shown in Figure 2 except for the lower temperature. Comparing Figure 6 with Figure 2, it can be observed that the curves in Figure 6 are almost the same as those in Figure 2, except that the curves change more sharply at the turning points in Figure 6 than those in Figure 2. This is because the Fermi distribution functions at lower temperature in Equations (7a)–(7d) change more steeply at the Fermi surface than that at higher temperature. (This is because the Fermi distribution functions in Equations (7a)–(7d) change more steeply at the Fermi surface when the temperature decreases).

**Figure 7:**
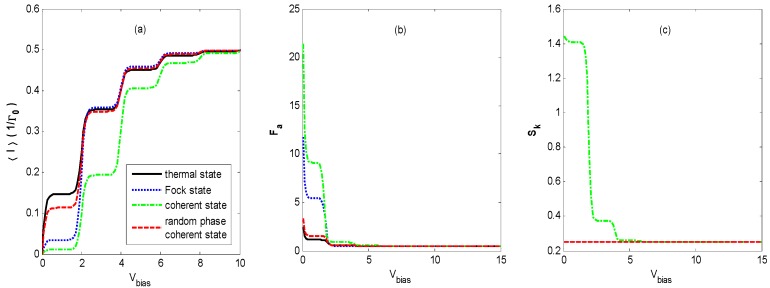
〈I〉 (**a**), *F_a_* (**b**), and *S_k_* (**c**) versus the bias voltage *V_bias_* for different states of optical field with the same parameters as Figure 2 except for the stronger electron–photon coupling coefficient, *λ* = 0.8. The skewness curves coincide for the thermal state, Fock state, and coherent state with random phase.

Figure 7 shows the FCS properties of QD charge transport versus the bias voltage *V_bias_* for different states of optical field for the same parameters as shown in Figure 2 except for the stronger electron–photon coupling coefficient. Comparing Figure 7a to Figure 2a, we find that more steps appear before the average currents reach saturation values as the bias voltage *V_bias_* increases for all of the different states of the optical field, because there are more photon sidebands entering the bias voltage window when the electron–photon coupling becomes stronger. 

Figure 7b demonstrates that the evolution of the Fano factor-voltage characteristic for the coherent state shows completely different behavior from that observed in Figure 2b. However, the changes observed in Figure 7b for the other states are similar to those of the corresponding curves in Figure 2b. The Fano factors decrease quickly to 0.5 in one step with increasing bias voltage *V_bias_*. The plateau values of the steps are obviously higher than those in Figure 2b, respectively, and avalanche-like transport also exists at a lower bias voltage. This is because more photon sidebands appear with an increasing bias voltage, and this enforces the effect of the dynamical channel blockade. 

Figure 7c illustrates that the skewness-voltage characteristics are similar to that in Figure 2c for all of the different states of the optical field, except there are higher plateau values of the steps, and there is one more step for the optical field in the coherent state as bias voltage *V_bias_* increases. Calculations also confirm the correlation between skewness and $\rho_{0}-\rho_{1}$.

**Figure 8:**
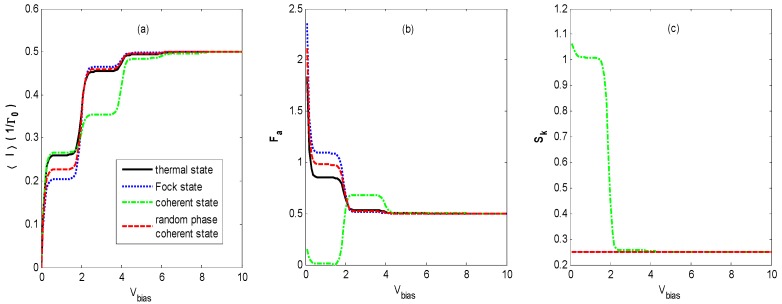
〈I〉 (**a**), *F_a_* (**b**), and *S_k_* (**c**) versus the bias voltage *V_bias_* for different states of optical field with the same parameters as Figure 2 except for the stronger light intensities, *N_th_* = *N_Fock_* = $\alpha^{2}$ = 2. The skewness curves coincide for the thermal state, Fock state, and coherent state with random phase.

Figure 8 shows the FCS properties of QD charge transport versus the bias voltage *V_bias_* for different states of optical field with the same parameters as Figure 2 except for the stronger light intensities. It illustrates that the FCS properties of QD charge transport behave similarly to those in Figure 2 besides the different shoulder heights of the steps.

**Figure 9:**
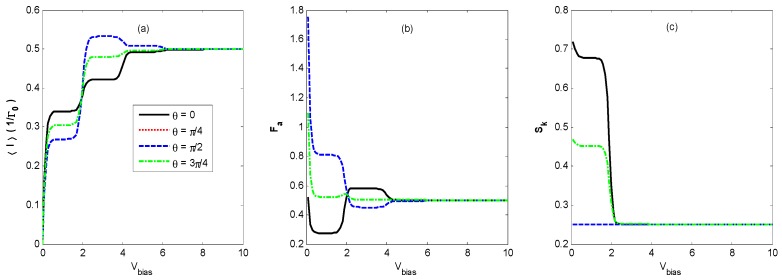
〈I〉 (**a**), *F_a_* (**b**), and *S_k_* (**c**) versus the bias voltage *V_bias_* for the coherent state with different phases for the same parameters as Figure 2. The curve of π/4 is the same as that of 3π/4.

Figure 9 exhibits 〈I〉, *F_a_*, and *S_k_* versus the bias voltage *V_bias_* for the coherent state with different phases. It shows that the phase of the coherent state can evidently change the FCS property: In particular, negative differential conductance appears at a bias voltage of nearly 4 and 6 in Figure 9a. As for the other states, the thermal state and coherent state with random phase are mixed states, and the phase of the Fock state is completely uncertain. Here, only the coherent state optical field has the advantage of tuning the FCS properties by phase. The interaction between the QD and coherent state optical field can lead to the appearance of obvious negative differential conductance in a symmetric QD system, while the pronounced negative differential conductance, which usually appears in a similar QD system assisted by phonons with asymmetric parameters and negative differential conductance, is ordinarily much less pronounced in symmetric junctions [9,40]. 

**Figure 10:**
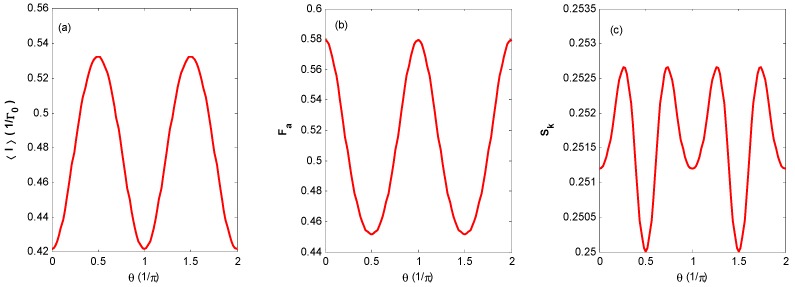
〈I〉 (**a**), *F_a_* (**b**), and *S_k_* (**c**) versus phases of the coherent state with the same parameters as Figure 2.

Figure 10 exhibits 〈I〉, *F_a_*, and *S_k_* versus the phase of the coherent state with fixed bias voltage, *V_bias_* = 3. From Figure 10, we can find that 〈I〉, *F_a_*, and *S_k_* can be changed by a period of π for the phase of the coherent state. Although the definite phase of the optical field is unmeasurable, this result implies that the FCS properties can be periodically changed by tuning the relative phase of the laser beam.

**Figure 11:**
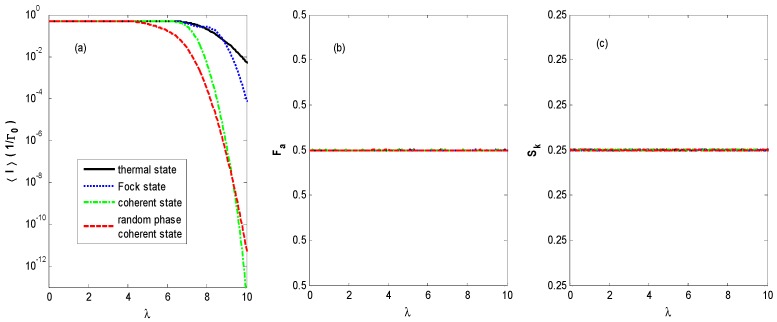
〈I〉 (**a**), *F_a_* (**b**), and *S_k_* (**c**) versus electron–photon coupling constant λ for different states of optical field with the same parameters as Figure 2 except for the infinitely high bias voltage.

Finally, Figure 11 demonstrates the FCS properties of QD charge transport versus the electron–photon coupling coefficient under infinitely high bias voltage. Figure 11 shows exponentially suppressed average currents for the different states of optical field due to the very strong electron–photon interaction. The Fano factor and skewness always stay within the constant value dominated by the Coulomb repulsion and the Pauli exclusion principle, because all the photon sidebands are in the window of bias voltage.

## 4. Conclusion

In summary, we employed the particle-number-resolved master equation and took into account the influences of optical fields in the thermal state, Fock state, coherent state, and coherent state with random phase. Then, we investigated the FCS properties of single-electron tunneling transport on a QD in a moderate electron-photon interaction regime by single-particle approximation. The results show that the interaction with the optical fields in different quantum states can lead to critical effects on the average tunneling current, Fano factor, and skewness of single-electron tunneling transport on the QD. 

Firstly, under the same light intensity, the average tunneling currents have different shoulder heights for all of the studied quantum states of optical fields when the bias voltage *V_bias_* is lower, while they have the same saturation current as the bias voltage *V_bias_* becomes higher. The vacuum state optical field can unexpectedly induce a staircase-shaped current. For the coherent state, the characteristics of the Fano factor and skewness are different from those of all other states.

Secondly, the FCS properties are sensitive to the energy level of the QD, the electron–photon coupling coefficient, and the phase of the coherent state for single-electron tunneling transport on a QD. With regard to avalanche-like transport at a lower bias voltage, the mechanism in a moderate electron-photon interaction regime is the dynamical channel blockade, which is different from that for a strong electron–photon interaction regime. However, the Fano factor voltage characteristics are dominated by the Pauli blockade and Coulomb blockade at higher bias voltages.

Thirdly, pronounced negative differential conductance results from tuning the phase of the coherent state optical field in a symmetric QD system, while the pronounced negative differential conductance usually appears in a similar QD system assisted by phonons with asymmetric parameters. The FCS properties vary with the phase of the coherent state in a π-period. 

Finally, under infinitely high bias voltages, the evolution behaviors of the average current diverge at higher electron–photon coupling coefficients for the different states of optical field.

On the whole, for single-electron tunneling transport on a QD, the FCS properties can be strictly regulated by the optical field under different quantum states, especially by the phase of the coherent state and QD energy level adjusted by gate voltage. Thus, the investigation is significant for controlling the transport of a QD electron for nanostructure devices.
